# Opening Pandora’s box - key facilitators of practice change in detecting and responding to childhood adversity - a practitioner perspective

**DOI:** 10.1186/s12887-024-04918-5

**Published:** 2024-07-18

**Authors:** Sarah Loveday, Lingling Chen, Leanne N. Constable, Ashraful Kabir, Natalie White, Sharon Goldfeld, Lena Sanci, Harriet Hiscock

**Affiliations:** 1 Department of Paediatrics, University of Melbourne, Parkville, VIC Australia; 2 Health Services, Murdoch Children’s Research Institute, Melbourne, VIC Australia; 3 Policy and Equity, Murdoch Children’s Research Institute, Melbourne, VIC Australia; 4 Centre for Community Child Health, The Royal Children’s Hospital, Melbourne, VIC Australia; 5 Department of General Practice, University of Melbourne, Parkville, VIC Australia

**Keywords:** Practice change, Childhood adversity, Integrated care, Qualitative research

## Abstract

**Background:**

Childhood adversities worsen physical and mental health across the lifespan. Health and social care practitioners play a key role in identifying and responding to childhood adversity, however, may be reluctant to do so due to a perceived lack of services to refer to, time pressures and a deficit of training and confidence. We aimed to (1) quantify changes in practitioner comfort and confidence to identify and respond to childhood adversity following a multimodal intervention within an integrated child and family health and social care hub and (2) to understand barriers and facilitators of practice change.

**Methods:**

Hub practitioners were surveyed about their competence and comfort to directly ask about and confidence to respond to adversity at baseline and then at six and twelve months post training. Interviews were undertaken to explore practitioner barriers and enablers of practice change. Interviews were recorded, transcribed verbatim, and analysed using reflexive thematic analysis. The theoretical domains framework was used to identify the key drivers of practice change.

**Results:**

Fifteen of 18 practitioners completed all three surveys and 70% reported increased competence and comfort to directly ask, and confidence to respond across a range of adversities over the 12-month intervention. Twenty-one practitioners completed interviews. Six themes were identified as either facilitators or barriers to practice change. Facilitator themes included (1) connection matters, (2) knowledge provides assurance, (3) confidence in ability and (4) choosing change. Barrier themes were (1) never enough time and (2) opening Pandora’s box. Following analysis, key drivers of practice change were ‘social influence’, ‘belief in capability’, ‘knowledge’ and ‘behaviour regulation’ while barriers to practice change were ‘environmental context and resources’ and ‘emotion’.

**Conclusions:**

Practitioners reported improved confidence in identifying and responding to adversity through a multimodal intervention delivered in an integrated Child and Family Hub. Changing practice requires more than just education and training. Opportunities for social connection and coaching to improve self-confidence and perceived competence are needed to overcome the fear of opening Pandora’s box.

**Supplementary Information:**

The online version contains supplementary material available at 10.1186/s12887-024-04918-5.

## Introduction

Childhood adversity encompasses a range of events or stressors that occur within the child’s family or social environment and have a negative impact on physical and mental health across the lifespan [1–3]. These include child abuse and neglect, parental mental health difficulties, family violence, parental drug and alcohol abuse, housing instability, food insecurity, victimisation, and discrimination [2–4]. The burden of childhood adversity is significant with 30% of all mental health disorders attributable to childhood adversity including 30% of all anxiety disorders, 40% of depression and 67% of lifetime suicide attempts [5, 6]. Furthermore, childhood adversity is common, with 52.8% of Australian children experiencing two or more adversities by their 11th year with an unequal distribution of adversity [7]. Children who have a low socioeconomic position or are indigenous or from an ethnic minority are 4–8 times more likely to experience adversity compared with wealthy children from Anglo-Euro backgrounds [7]. 

Identification of childhood adversity is the first step in being able to respond and potentially change outcomes, but there is debate as to how adversity should be identified, either in the context of a routine encounter with a health care practitioner or in broader population screening [8–11]. Practitioners recognise the importance and value of identifying adversity with most practitioners supporting routine identification of social needs [12–14]. However, practitioners are reluctant to ask about adversity due to a perceived lack of community services to refer to, time pressures and a deficit of training and confidence to respond once adversity is identified [15]. In addition, practitioners report feelings of discomfort, fear, and anxiety in asking about adversity which influences their practice and makes them less likely to address adversity in consultations [16–19]. Improving practitioner confidence and comfort to ask about adversity will likely be necessary to achieve systematic identification of childhood adversity in health care settings.

Moreover, identifying childhood adversity routinely during standard encounters will require a substantial change in practice. Practitioners have low rates of asking about adversity with only 9–30% of practitioners routinely asking even when personally motivated [19, 20]. Practice change is difficult and requires sustained motivation and support [21]. It has been recognised that there is “no magic bullet” for achieving practice change [22]. Single interventions such as education alone are rarely effective in achieving sustained practice change and yet these are the most commonly employed in health care interventions [23, 24]. Understanding the barriers and facilitators of practice change using behaviour change theory is critical to achieving sustained change [25, 26]. Much of the literature on practice change has been geared towards practitioners using evidence-based practice or guidelines. There are no studies to date that use a theoretical model to understand the facilitators of practice change across a range of health and social care practitioners in improving identification and response to childhood adversity.

We know from previous research that practitioners do not have the training, or confidence to ask about adversity, and that they have challenges engaging with families and connecting families to appropriate community resources due to lack of knowledge of local services [15]. Overcoming these barriers to improve practitioner identification and response to childhood adversity will require a change of practice. We therefore aimed to, in a sample of health and social care practitioners working in an integrated Child and Family Hub(CFH) [27] (i) quantify changes in practitioner comfort and confidence to identify and respond to childhood adversity following a multimodal intervention and (ii) to understand barriers and facilitators of practice change across a range of practitioners.

## Methods

This study used a concurrent triangulation mixed methods design with quantitative and qualitative data collected and analysed concurrently and triangulated [28]. Qualitative data was used to validate and explain quantitative data regarding practitioner change in identifying and responding to adversity and to highlight key facilitators and barriers of practice change.

### Study setting

This study was undertaken as part of a broader study to implement an integrated CFH at a community health centre (IPC Health) Wyndham [27]. Wyndham is a culturally diverse local government area of Greater Metropolitan Melbourne, Australia with high levels of immigration. It has known population risk factors for adversity with higher rates of unemployment, housing stress and social isolation compared to greater Melbourne [29, 30]. IPC Health Wyndham Vale is a community health centre and general practice clinic that provides primary medical care, community paediatrics, allied health, and dental care.

**Fig. 1:**
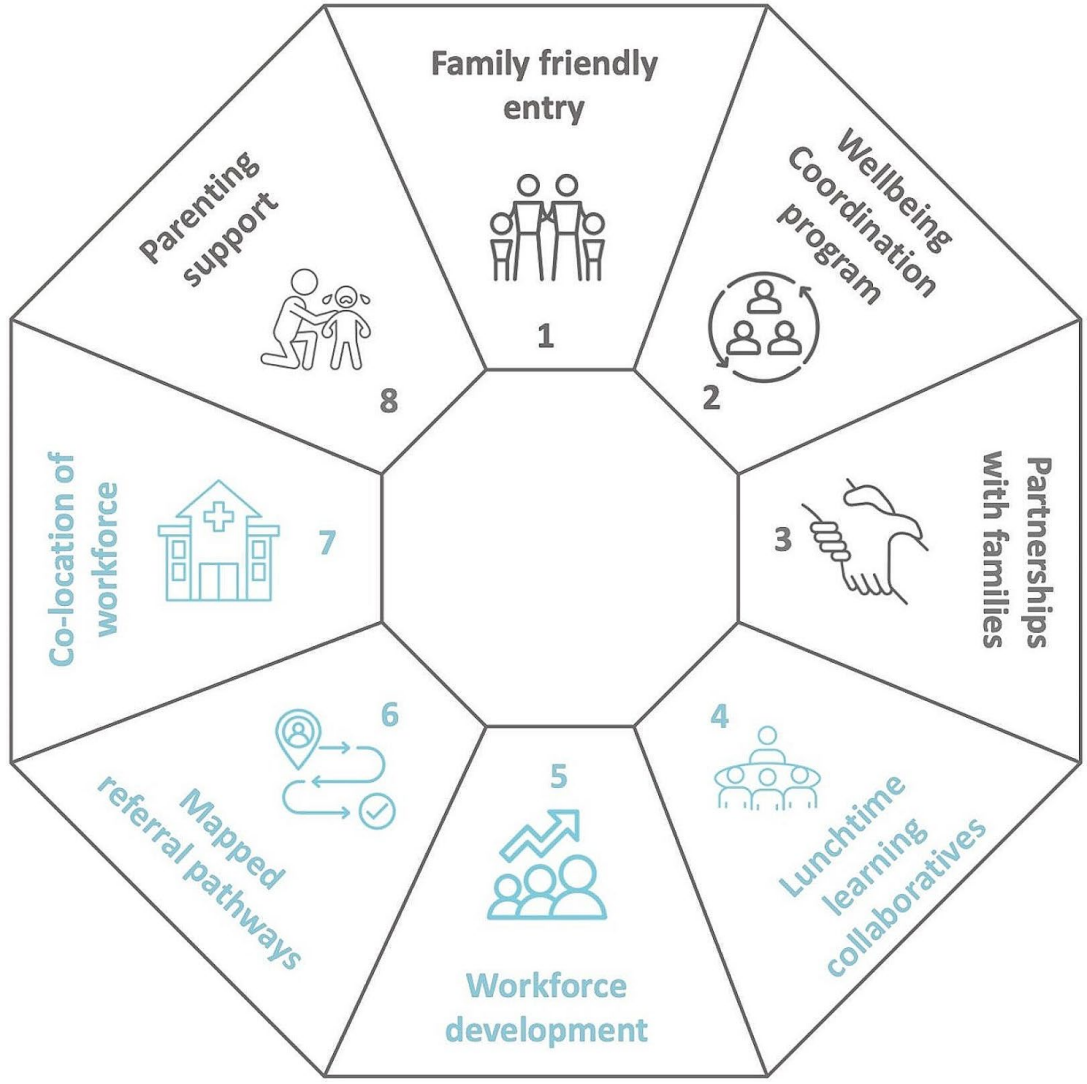
The child and family hub model

The CFH is an integrated care initiative which aimed to improve child mental health through better identification and response to adversity for families of children aged 0–8 years old. The wider project aimed to codesign, test, and evaluate integrated CFH models over two sites and to examine the impact of the CFH on caregiver reported identification of adversity and referrals to services [27]. The CFH is a co-designed model of care which integrates health and social care through eight separate components as seen in Fig. 1 [27, 31]. 

Four components aimed to improve family engagement and access to care encompassing a “no-wrong-door” approach with caregivers being asked about adversity and provided support by all practitioners within the CFH, parenting support delivered by practitioners in the CFH, partnership with families to strengthen the connections to the community and a wellbeing coordination program to identify the holistic needs of the family and to help connect families to community services. The other four components were directed at practitioners within the CFH. Practitioners were trained using principles of Family Partnership Model to improve engagement with families and the Parent Engagement Resource (PER) [32] which assists practitioners to directly ask about adversity. Mapped referral pathways provided practitioners with a community directory of local services to increase practitioner knowledge and confidence to respond to adversity. Monthly learning collaborative meetings were designed to imbed training and to provide a community of practice. Finally, co-location of services enabled the development of ‘warm referral’ pathways [27, 33]. 

### Conceptual framework

The Theoretical Domains Framework (TDF) and the COM-B model of behaviour change (COM-B) were used to guide the development of this study. Behaviour can be understood as the result of interactions between capability, opportunity, and motivation. Capability encompasses both physical and psychological capability while opportunity encompasses all the factors that work outside of an individual to either promote or inhibit behaviour [34]. Motivation is the internal processes which stimulate and direct behaviour [34]. Achieving behaviour change requires a change in opportunity and capability which in term improve motivation and results in change.

The TDF integrates and simplifies 33 theories of behaviour change into a framework with 14 domains. Determinants of behaviour encompass individual, social and environmental factors with the majority of domains related to individual capability and motivation. Each domain has several related behavioural constructs; for example, belief about capabilities encompasses the constructs of self-confidence, perceived competence, self-efficacy, perceived behavioural control, beliefs, self-esteem, professional confidence and empowerment. The TDF has been used across a range of disciplines and situations to provide a better understanding of facilitators and barriers to behaviour change [35]. Huijg et al. developed a validated TDF questionnaire that discriminately measure the majority of TDF domains to determine practitioner implementation behaviours [36]. 

**Fig. 2:**
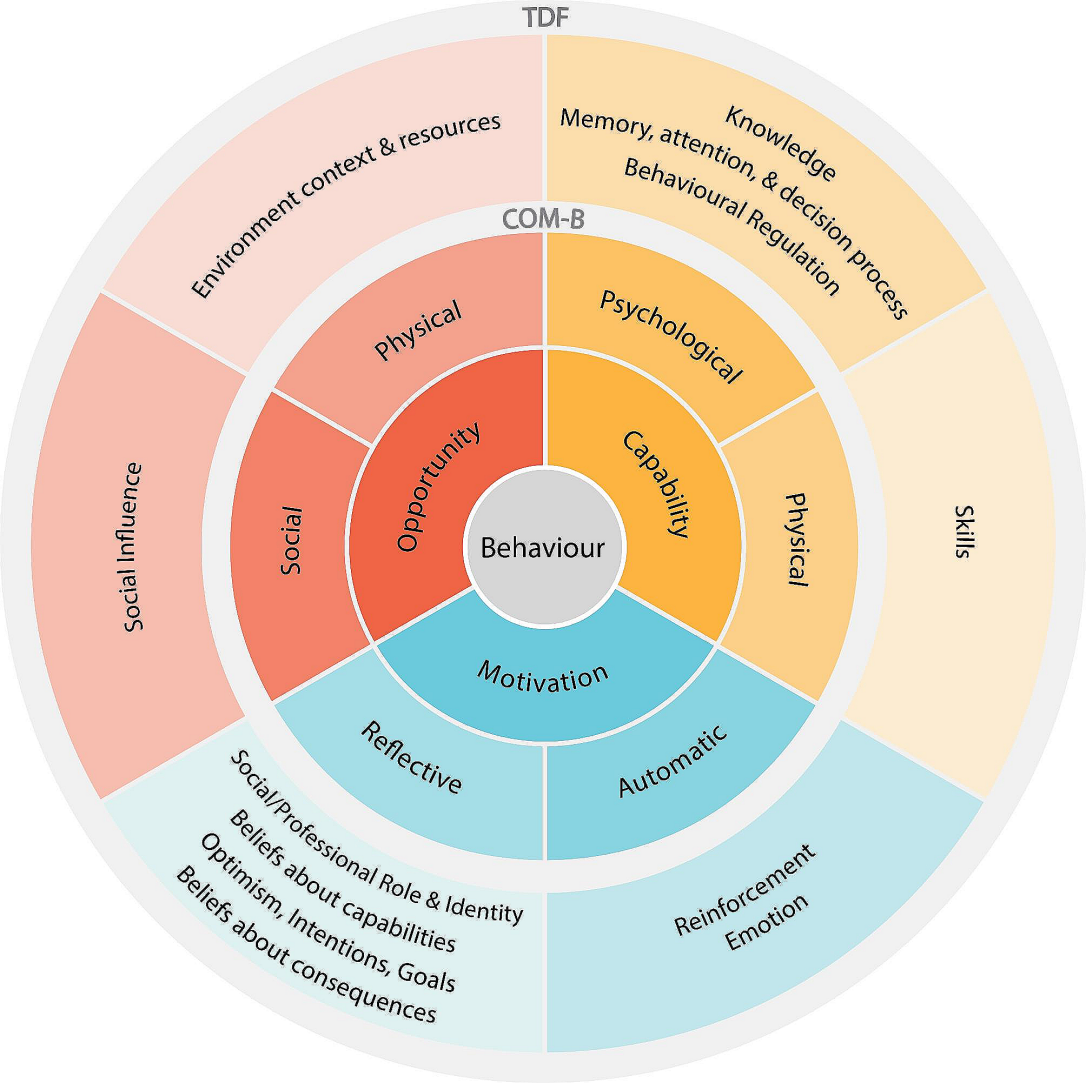
The relationship between the theoretical domains framework and behavioural change wheel adapted from Atkins et al. [35].

The TDF has been linked to the COM-B model and expands on the psychological capability and reflective motivation constructs of the COM-B so enabling a greater understanding of the behavioural targets to induce behaviour change as demonstrated in Fig. 2.

### Participants

Practitioners who worked across health (general practitioners, paediatricians, allied health practitioners, nurses) and social care (financial counsellor, wellbeing co-ordinator and lawyers) at IPC Health were invited to take part in the implementation of the CFH [33]. Twenty practitioners were approached and invited to take part with two declining to participate in the CFH due to work pressures (general practitioner and community nurse). Participation in the CFH involved a day-long training session to improve practitioner identification and response to childhood adversity and attendance at monthly learning collaborative meetings. Practitioners were also provided with new services within the CFH (legal, financial counselling and wellbeing coordinator) as well as mapped referral pathways for community resources [27]. 

There were some changes to practitioner involvement over the 12 months due to external staffing changes. All three lawyers who joined at the beginning of the CFH changed due to external commitments with two lawyers taking on an overseeing role of other lawyers from their organisation. Two other practitioners left their roles due to changes in employment, one at 4 months and one at 11 months. In addition, two new practitioners joined the CFH (practice nurse and maternal child health nurse) at 9 months. All practitioners who had completed training and had more than 9 months of involvement in the CFH were eligible to participate in the 12-month survey (*n* = 16). Practitioners who had completed training and were actively involved in the CFH at the end of the 12-month period were eligible to participate in an interview (*n* = 21). This included new CFH practitioners (legal and nursing) (*n* = 5) in addition to original CFH practitioners (*n* = 16).

Written consent and demographic information were collected at the start of the study prior to the implementation of the CFH.

### Child and family hub multimodal intervention

A multi-modal approach to encourage practitioner behaviour change as outlined in Table 1. A range of behavioural change techniques as defined by Michie et al. were employed which mapped onto different TDF domains [26]. These included goal setting, social support, role play, homework tasks, instruction and training and providing prompts. Practitioners were supported to change practice through attending a one-day training session and then had regular coaching and support in a community of practice (lunchtime learning collaborative). Practitioners were encouraged to directly ask about adversity, however, this was adapted to clinical practice to not burden practitioners who had limited time e.g. practitioners might ask about one type of adversity during a clinical encounter. Additionally, referral pathways for responding to adversity were improved as described above as part of the CFH.

**Table 1:** Behavioural change techniques as defined by Michie [26]

Behavioural Change Techniques	Definition	Mapped to TDF Domain	CFH Intervention Example
Goal	Set behavioural goal	Skills Goals Intentions	Practitioners were encouraged to set goals as part of a reflective practice exercise at 6 and 9 months. In addition, there was a goal setting exercise at month 11.
Goal review	Assess extent to which goal is achieved and identify factors influencing this	Skills Goals Intentions	Practitioners reviewed their goals at three separate time points. Barriers to asking about adversity were discussed.
Social support	Others listen, provide empathy and give generalised positive feedback	Social influence	The monthly learning collaboratives provided social support
Social comparison	Provide opportunities for social comparison	Social influence	Practitioners shared their experiences of asking about adversity during learning collaboratives
Set homework tasks	Practice behavioural tasks	Skills	Practitioners given specific homework following learning collaboratives i.e. to ask 1 family each day at least 1 direct question about adversity
Role play	Perform behaviour in simulated situation	Social influences Social /Professional Role and identity	As part of training, practitioners had the opportunity to practice directly asking one another questions about adversity
Instruction	Teach new behaviour required for performance of target behaviour	Knowledge Skills	Practitioners were trained to ask about adversity through initial training and then through coaching each month
Prompt	Stimulus that elicits behaviour	Attention, memory and decision processes	Posters and postcards were co-designed with practitioners to help prompt change in behaviour

### Data collection

#### Quantitative data

Practitioners were asked to complete three online surveys hosted through the secure online electronic data program (REDCap) [37] at set time points over a year (baseline, 6 months, and 12 months post training) as well as participate in an interview at 12 months. Practitioners were asked about their experience of identifying and responding to adversities across three broad domains; outside the home, inside the home and broader social adversities using the expanded definition of adversity from Karatekin and Hill [4] as seen in Table 2 which widens the original definition of adverse childhood experiences to include social adversities [2]. 

**Table 2:** Adversity domains from practitioner questionnaires

Adversities outside the home	Adversities inside the home	Broader social adversities
Social support	Physical health and disability	Visa or migration challenges
Financial challenges	Parental mental health challenges	Discrimination or harassment
Housing challenges	Parenting challenges	Criminal justice involvement
Employment issues	Family relationship challenges	
	Family violence	
	Drug and alcohol abuse	
	Child abuse and neglect	
	Child neglect	

Practitioners were asked to rate their competence and comfort to directly ask about each adversity and their confidence to respond to each adversity listed in Table 2 (*n* = 15) using a study-designed 5-point Likert scale (from (1) not at all to (5) extremely). The concepts of competence and confidence are not synonymous but are closely linked. Confidence has been defined as “a belief in one’s own abilities or qualities” [38] whereas competence is defined as “the ability to do something successfully or efficiently” [39]. Comfort is described as “a psychological state wherein a person is at ease and in control of their environment experiencing low levels of anxiety and stress” [40]. 

In the final survey at 12 months, practitioners were asked to rate their agreement for a series of statements from a TDF questionnaire developed from Huijg et al [36] using a 5-point Likert scale (from (1) strongly disagree to (5) strongly agree) to identify key factors influencing practitioner behaviour.

#### Qualitative data

Interviews focused on facilitators and barriers to individual practitioner practice change. The interview guide was developed through discussion with the research team (see supplementary material). Practitioners were asked about their experience of directly asking about adversity and responding to adversity and their experience of being involved in the CFH.

In-depth semi-structured individual (*n* = 18) and group (*n* = 1 group) interviews were conducted with 21 practitioners who participated in the implementation of the CFH. The group interview with the practice nurses (*n* = 3) was conducted due to time constraints of the participants as well as enabling a discussion of their shared learning and experiences because they work closely as a team. The interviews were conducted by two members of the research team (SL, LNC) between March 2023 and May 2023 averaging 39 min (range 29 –70 min). Interviews were conducted in person at IPC Health, or via video conferencing platforms (Zoom or Microsoft Teams) or telephone according to participant preference.

### Data analysis

#### Quantitative data analysis

Practitioner data reporting competence, comfort, and confidence was dichotomized with ‘extremely’ and ‘good’ considered a positive result. The proportion of positive results was then converted to a percentage. Formal statistical analysis was not conducted due to the small number of participants (*n* = 16). We used the definitions for reporting dichotomous outcomes as proposed by Grimshaw [41] with ‘Small’ to describe effect sizes ≤ 5%; ‘Modest’ to describe effect sizes > 5% and ≤ 10%; ‘Moderate’ to describe effect sizes > 10% and ≤ 20%, and ‘Large’ to describe effect sizes > 20%.

The TDF domains that were endorsed were determined as having ≥ 50% practitioner agreement using the highest point on the Likert scale (strongly agree).

#### Qualitative data analysis

Interviews were analysed using reflexive thematic analysis. The analysis followed the six-step process as described by Braun and Clarke (2019); (1) familiarisation with the data, (2) coding, (3) generating initial themes, (4) renewing themes, (5) defining and renaming themes, and (6) writing up [42]. 

The interviews were recorded, transcribed, and edited for clarity and imported into NVivo for analysis. Transcripts were triangulated with field notes (SL, NW) and interview notes (SL, LNC) to improve credibility of findings and to confirm interpretations made while reading the transcripts.

Initial coding of transcripts was inductive and reflexive with keywords or phrases assigned a code using NVivo 14. Transcripts were coded independently by three members of the research team (SL, AK and LC), with all transcripts dual-coded by two researchers. Identified codes were discussed to ensure clarity of meaning and included if there was consensus. Following the completion of the initial coding, two members of the research team (SL, LC) grouped codes into categories and then developed themes using both inductive and deductive analysis. Codes that did not relate to either barriers or facilitators of practice change were not included in the final analysis. Themes were discussed and iterated until overarching themes were identified that related to practice change. The final themes were cross referenced with the TDF to determine the most important facilitators and barriers to practice change. These themes were then tested against each transcript to ensure validity. The analysis was an iterative process with discussion at each point to resolve any conflicts and to clarify thematic development.

A decision was made not to record the professional discipline of individual participants because of the potential for identification, however, differences across professional groups and types were analysed and reported on. Quotes were labelled with participant number and broad grouping (health or social care practitioner).

### Researcher positions

Four members of the research team are medical doctors (SL, SG, LS, HH); the other members include a psychology researcher with expertise in qualitative research (LC), a researcher in public health with experience in qualitative research (AK), a lived experience researcher (LNC) and a senior research officer (NW). Three members of the research team (SL, LNC, NW) were involved in the implementation of the CFH which provided a deeper understanding of the barriers and facilitators the practitioners experienced.

## Results

### Participant demographics

Eighteen practitioners were enrolled prior to implementation of the CFH. Most practitioners who took part were female (> 85%) and had different roles across health care (paediatricians, general practitioners, nurses, and allied health practitioners) and social care (social workers, lawyers, and financial counsellor) as shown in Table 3. Seventeen practitioners completed surveys at baseline and 6 months and 15 practitioners completed the survey at 12 months, with an overall response rate of 96%. Twenty-one practitioners participated in the interviews. There were more health care practitioners (*n* = 13) than social care practitioners (*n* = 8) who participated in an interview. The two groups of practitioners were similar in age range and number of years in the role.

**Table 3:** Practitioner demographics

Practitioner Characteristics	*n* (%) *n* = 18 Practitioners at Baseline	*n* (%) *n* = 21 Participated in interviews
Age		
18–24 years	1 (5.5)	1 (4.8)
25–34 years	3 (16.7)	3 (14.3)
35–44 years	7 (38.9)	9 (42.8)
45–54 years	3 (16.7)	4 (19.0)
55–64 years	3 (16.7)	3 (14.3)
65–74 years	1 (5.5)	1 (4.8)
Number of years in role		
< 2 years	2 (11.1)	2 (9.5)
3–5 years	5 (27.8)	6 (28.6)
6–10 years	5 (27.8)	6 (28.6)
> 10 years	6 (33.3)	7 (33.3)
Service provider gender		
Female	16 (88.9)	18 (85.7)
Role		
Paediatrician/Paediatric Fellow	3 (16.7)	3 (14.3)
General Practitioner	2 (11.1)	2 (9.5)
Nurse (MCHN, Practice Nurse)	5 (27.8)	6 (28.6)
Allied Health (Speech Pathologist, Dietician)	2 (11.1)	2 (9.5)
Financial Councillor	1 (5.5)	1 (4.8)
Lawyer	3 (16.7)	5 (23.8)
Social Worker	2 (11.1)	2 (9.5)

*MCHN = maternal child health nurse

### Practitioner competence, comfort and confidence

**Fig. 3:**
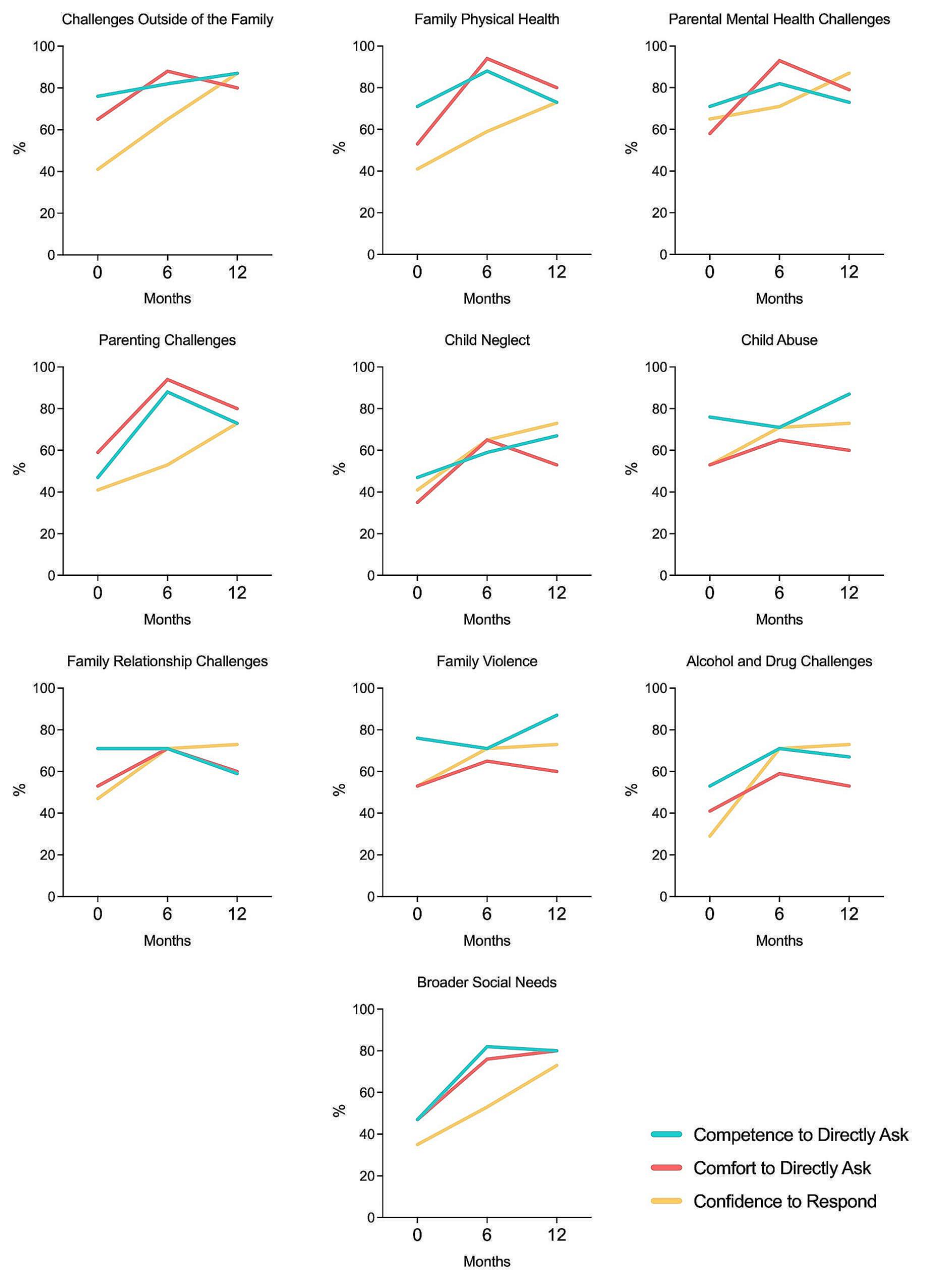
Changes in practitioner self-reported competence, comfort and confidence

Practitioners’ competence and comfort to directly ask about adversity and their confidence to respond improved over the 12 months of the study as demonstrated in Fig. 3. Practitioner self-reported competence to directly ask about adversity had a modest increase over all types of adversity except family relationship challenges, with an average percentage change of 9.7% (range 11–26%). There was a moderate improvement in self-reported comfort to directly ask about all types of adversity with an average percentage change of 19.2% (range 7–32%). Practitioner self-reported confidence to respond to adversity demonstrated a large improvement with an average percentage change of 33.1% (range 20–46%). The largest improvement was seen in practitioner confidence to respond to adversities outside of the home which likely reflects the new practitioner roles and referral pathways that were co-located in the CFH particularly financial counselling, wellbeing co-ordination and legal support. Practitioner levels of comfort lagged behind their self-reported competence for directly asking about some adversities, namely family violence, alcohol and drug challenges and child abuse and neglect. Despite practitioners reporting that they were competent, their reported comfort to directly ask about these adversities only increased by a small degree over the 12 months. Moreover, for all types of adversity, practitioners’ comfort peaked at 6 months but decreased again at 12 months despite an overall improvement in practitioner confidence and competence. This change may reflect the challenge to overcome practitioner fear of directly asking about adversity leading to a dip in practitioner comfort.

### Thematic analysis

There were six themes identified during qualitative analysis. Four themes were facilitators of practice change (1) connection matters, (2) knowledge provides assurance, (3) confidence in ability and (4) choosing change. Two themes were barriers to practice change (5) never enough time (or money) and (6) opening Pandora’s box.

#### Theme 1: connection matters

Practitioners discussed the importance of connection with each other to improve practitioners’ confidence to identify and respond to adversity. Connection was seen as a key driver of practice change for both health and social care practitioners. This social connection was delivered through the community of practice which enabled opportunities for peer coaching and support.

Listening to each other and learning from each other were facilitators of practice change with practitioners being more motivated to ask about adversity when they felt more supported.

*I feel like it’s been fantastic in having that time and space to build networks and to support each other with asking about adversity. So*,* professionals could bounce off each other with how they can ask questions or how they could refer families because the services system is always changing. (P16*,* social care practitioner)*.

Working in a team improved practitioners’ enjoyment of their role and provided emotional support because working with adversity was seen as emotionally taxing. Social connection was an important counter to practitioner burnout.

*I enjoy being part of a team. And that’s how I felt*,* that I wasn’t sitting out there on my own*,* that I was part of a team. And I think when*,* because it’s often heavy*,* like it’s heavy and knowing that that weight is shared collaboratively in terms of that responding to adversity makes the work*,* well*,* the work is meaningful anyway*,* but it felt like it was meaningful that you were working together as a team*,* and so because of that I enjoy coming to my job. (P3*,* health practitioner)*.

Connection between different practitioners was facilitated by regular meetings, with practitioners across all disciplines seeing this as critical in developing a team approach. The more regular the attendance at meetings the “more practitioners got out of it”(P17).

*I thought that it was really good that the monthly meetings were set up. Attendance at those was really the core of how practitioners were getting together and talking to each other and connecting and learning about what one another did. I think without those monthly meetings it probably wouldn’t have worked. (P20*,* social care practitioner)*.

Practitioners discussed wanting more connection and reflected on the barriers to connection being the physical building and part-time work.

*It’s so much nicer to be able to just go up to someone and ask them a quick question if you need to. I’m aware of where the wellbeing coordinator sits. Then everybody else seems to be floating around. They probably think that about me as well because we’re never onsite at the same time*,* so I think that’s tricky. I think having that more of that cohesion in one space*,* there’s more of a team collaboration. (P5*,* health practitioner)*.

#### Theme 2: knowledge provides assurance

Knowledge of services was a key facilitator of practice change with practitioners across all disciplines reporting greater confidence to respond to adversity by knowing that the services were available and how to access them.

*I think knowledge [of services] makes you feel more confident. Without knowledge it’s like…we can answer your question*,* but what advice are we going to give you? (P7*,* health practitioner)*.

While knowledge of services in the community and within the CFH was very important, practitioners all talked about knowing “the person” and that being able to make a “warm referral” was even more important for changing their practice. Knowing who to refer to and how to respond to adversity meant that practitioners were more likely to ask about adversity.

*And the reason I think that I felt comfortable doing that is because firstly*,* I felt comfortable identifying adversity*,* but secondly*,* because I knew the team supporting. (P3*,* health practitioner)*.

*We’re all out there now a little more educated and a little more confident in knowing where to make appropriate referrals*,* which then leads you to being a little more confident to have those discussions in the first place. (P17*,* health practitioner)*.

Practitioners recognised that understanding other practitioners’ roles leads to greater confidence to refer. Both knowledge of services and knowing other practitioners and their roles in supporting families with adversity made responding to adversity less daunting for practitioners.

*Having this in the handbook*,* having referral forms handy to quickly fill out and send through to the right person that made the work much more efficient*,* and not*,* to be honest*,* not so daunting thinking about where do I actually now refer this family when I find adversity. (P2*,* health practitioner)*.

Practitioners also reported that families were more likely to take up referrals if they trusted that the practitioner had knowledge of the service and the person they were being referred to.

*Being able to say*,* hey*,* we’ve got this person attached to the Hub. I can put in a referral for you. It’ll be really easy. That has been fantastic*,* and I think clients have appreciated that as well*,* and for you to be able to say yes*,* you know this person*,* they sit next to me … that you actually know the person that they’re being referred to*,* and if they trust you as a worker*,* they trust that you are going to refer them to somebody appropriate. (P9*,* social care practitioner)*.

#### Theme 3: confidence in ability

Practitioners’ belief in their ability to identify and respond to adversity influenced their behaviour. Some practitioners had confidence in their ability to identify adversity due to prior training, role, and experience. This confidence in their ability was seen across both health and social care practitioners, however, was more common in social care practitioners.

*But I’m not afraid to ask about drug and alcohol*,* gambling*,* finances*,* their past trauma*,* their experiences in childhood*,* mental health*,* physical health*,* family violence*,* it’s just our bread and butter. (P14*,* health practitioner)*.

For other practitioners this confidence in their ability grew over time through training and support in the CFH. As practitioners became more confident and comfortable asking about adversity, they were more likely to change their practice and ask more frequently.

*Yes*,* you know*,* and I would say that the confidence*,* of course*,* grew with each meeting*,* each training*,* each help. (P4*,* health practitioner)*.

*I think I felt*,* comfortable is probably the word*,* just more and more comfortable in responding to adversity*,* acknowledging it*,* and empathising with the family*,* but also as part of that*,* being able to offer that I would be able to refer to the relevant professional. (P3*,* health practitioner)*.

The language that practitioners used influenced their confidence. Health practitioners grew more confident in their ability to identify adversity when they were more confident using language around adversity and had time to practice. This belief in their capability was a driving factor whether practitioners asked families about adversity.

*A big part of it is getting the language right in bringing up sensitive topics and asking about these things. Having the right language and asking in the right way is so important. And feeling confident with your own ability*,* your own language*,* and your own ability to go there. (P17*,* health practitioner)*.

#### Theme 4: choosing to change

Some practitioners made deliberate choices to support changing their practice. Practitioners discussed how the CFH helped them to become more aware and mindful of their own practice. This helped them to make changes as they were not operating on “automatic pilot”.

*It probably made me more conscious of seeking adversity rather than just letting them tell me about it. … And now I feel more empowered to go and seek it if it’s not volunteered. (P1*,* health practitioner)*.

*There’s that human habit of just going back to what you know. You’ve got a specific way of doing things and specific questions that you ask and things that you covered during your assessment*,* so if you’re not constantly being mindful to make an effort to ask these other additional things*,* it’s very easy just to keep slipping back into your old routine of how you conduct yourself. (P17*,* health practitioner)*.

Reflective practice was a key element of practitioners choosing to change and was seen across all practitioner types. The CFH enabled opportunities (i.e. lunchtime learning collaboratives) for reflective practice and the practitioners who were more engaged in this process were more likely to report that they had changed their practice in asking and responding to adversity.

*I think like the reflection has been good in relation to actively having a think back of certain times and what decisions we’ve been making and then coming back and seeing if that has changed. I think it’s good to reflect and something that I probably don’t do as much in practice*,* so it’s good to have that prompt to do so. (P22*,* social care practitioner)*.

*I mean it’s helped me in*,* I think*,* I’ve reflected in how I ask questions and what I do*,* so that’s been good. It’s given me another way to ask questions. (P15*,* health practitioner)*.

Some practitioners were surprised by the changes that the CFH had made but all wanted to continue to take these changes into their roles in the future.

*I want to tell you I have amended totally the way I am …. just come down a few octaves*,* and just see things how they see it. …It was a good process to go through*,* and … I didn’t join [the child and family hub] thinking that that would occur. I really didn’t*,* it just came*,* and it’s tempered me hugely. (P18*,* social care practitioner)*.

However, some practitioners did not feel that they needed to change and were less likely to be engaged overall. These practitioners had a negative experience of the CFH because “it ended up being different” (P15) than they had expected and the experience of being involved in the CFH was seen as “unhelpful” or “wasn’t necessarily relevant” (P16) as they felt that they were experts already.

*I did feel uncomfortable in the end because I’d spoken to our coordinator about not wanting to contribute … because I think it’s fantastic and it’s brilliant and I just felt like I was in the wrong place*,* if that makes sense*,* because I felt like I didn’t want to contribute because it’s what we do and my learning wasn’t going to be more than*,* it wasn’t something new to me. (P14*,* health care practitioner)*.

#### Theme 5: never enough time

Health practitioners discussed the main barrier to changing practice was time pressure. Practitioners reported wanting to ask but not feeling like they had the time within their normal appointments.

*We have very short appointments*,* like 10 minutes appointments*,* and sometimes like exploring those things*,* especially for example adversity*,* is something that needs a lot more time. But unfortunately*,* we do not have that time. Putting that question out and then not doing anything about it is even really worse. If we are putting that question out*,* time wise*,* what follow up can be arranged? (P13*,* health practitioner)*.

While this barrier was largely seen with health practitioners, some social care practitioners also commented on time pressures as having an impact on their practice.

*There is time constraints*,* so if it’s something that you’re not going to be able to do*,* like right now or within the time that the client needs*,* that’s when you try to find help. (P23*,* social care practitioner)*.

Both health and social care practitioners discussed funding as a barrier to changing appointment times or to changing the way they were able to respond to adversity.

*We unfortunately have in this bulk billing*^1^*clinic setup at IPC that we try to make it work financially for the community centre to be*,* so that we can actually continue the work and not have to cut back on appointment slots*,* it is an ongoing issue*,* that there is no funding for the service as such*,* and the service runs by the Medicare billings we make and…a small gap fee that we have. That’s a big issue I think. (P2*,* health practitioner)*

#### Theme 6: opening Pandora’s box

Like the mythical Pandora’s box, health and social care practitioners were fearful of directly asking about adversity in case they unleashed unforeseen problems. Practitioners were fearful that they would not be able to respond or that by asking about adversity they would damage their relationship with the family or cause harm.

*I think sometimes part of it can be not exactly knowing where to send people or what the answer should be. I guess like opening that can of worms and then being*,* oh look at the mess on the table type thing. And yes*,* I guess feeling like you’ve asked but then not been able to follow through. (P22*,* social care practitioner)*.

*And sometimes it is tricky because then you ask certain things and then you don’t get back. Your relationship can be stopped*,* because you know*,* we’re voluntary*,* they don’t have to use us. (P15*,* health practitioner)*.

If a family talked about having one adversity practitioners were reluctant to directly ask about others for fear of causing harm.

*But it’s also then tricky to go okay*,* we’re already know that you had housing instability we already know this*,* let’s add one more thing to make you feel crap about yourself. I guess I’m a little bit conscious about not trying to document too much adversity in some of my families. (P1*,* health practitioner)*.

This fear led to practitioners limiting both what adversities they asked about and the number of adversities they asked about to try to decrease the chance of having more unmet social needs that they can help with.

*I limit what I ask sometimes because as soon as you open the questions*,* you get Pandoras’ box. With these adversities*,* it’s just not one adversity. It’s 10*,* and then you have got a tackle; what you are going to do with ten adversities? (P21*,* health practitioner)*.

### Theoretical domains framework analysis

There were no domains that were significantly endorsed by practitioners demonstrating the complexity of identifying the key factors for changing behaviour. When a cut point of 50% was used, four domains were identified as outlined in Table 4. These included ‘professional role and identity’, ‘beliefs about capability’, ‘social influence’ and ‘environmental context and resources’.

**Table 4:** Practitioner endorsed statements from the theoretical domains framework

TDF Domain	TDF Statements	Agreement *N* = 14 *n* (%)
Knowledge	I am aware of the objectives of the CFH	6* (42)
Skills	I have the skills to directly ask about adversity	5* (38)
Social/professional role and identity	It is my responsibility to directly ask about adversity for families in my care	8 (43)
Beliefs about capability	I am confident that I can ask about adversity during my consultations even when there is little time	7 (44)
Optimism	I am optimistic about outcomes for families in the CFH when I ask about adversity	6 (45)
Beliefs about consequences	If I ask about adversity during my consultation, it will disadvantage my relationship with my clients	5 (36)
Intentions	I will definitely ask about adversity with my families in my next consultation	5* (38)
Environmental context and resources	There are good networks between practitioners in the CFH	7 (44)
Social influences	Most people whose opinion I value would approve of me asking about adversity with my clients	7 (44)
Memory, attention, and decision processes	How often do you forget to ask about adversity in your consultations	6 (45)

*Denominator is *N* = 13 due to missing data

Themes from the qualitative analysis were mapped onto the TDF domains and COM-B as seen in Table 5. The facilitators of practice change were social influence’ (e.g., social support and modelling), ‘knowledge’ (e.g., procedural knowledge), ‘belief about capabilities’ (e.g., self-confidence and perceived competence) and ‘behavioural regulation’ (e.g., making deliberate choices to change) and the main barriers were ‘environmental context and resources’ (e.g., time pressures and financial restraints) and ‘emotion’ (e.g., fear of negative outcome). Interestingly there were no themes that mapped to physical capability with psychological capability being a more important factor for driving practice change. Across health and social care practitioners there was agreement as to the most important facilitators of practice change.

**Table 5:** Themes mapped to the theoretical domains framework

COM-B Component		TDF Domain	Themes
Capability	Psychological	Knowledge	Knowledge Provides Assurance
		Memory, attention, and decision processes	
		Behavioural regulation	Choosing to Change
	Physical	Skills	
Opportunity	Social	Social Influence	Connection Matters
	Physical	Environmental context and resources	Never Enough Time (or money)
Motivation	Reflective	Social/Professional role and identity	
		Beliefs about capabilities	Confidence in Ability
		Optimism	
		Beliefs about consequences	
		Intentions	
		Goals	
	Automatic	Reinforcement	
		Emotion	Opening Pandora’s Box

## Discussion

This concurrent mixed methods study aimed to quantify changes in practitioner comfort and confidence to identify and respond to childhood adversity and then to identify the key barriers and facilitators of practice change. Practitioners were supported to change practice through a multi-modal intervention delivered through the CFH. Practitioners across health and social care all reported an improvement in competence, comfort and confidence to identify and respond to childhood adversity. Comfort to directly ask and confidence to respond improved more than competence which may reflect the high perceived competence at baseline where some practitioners already saw themselves as experts. Practitioners’ confidence to respond to adversities outside of the home (e.g. financial support, social support) had the largest change and may reflect the new referral pathways within the CFH. While knowledge improved practitioner confidence to respond to adversity, social connection and confidence in their ability were key drivers of practice change. Fear of ‘opening pandora’s box’ by directly asking about adversity was an important barrier and impacted on practitioner reported comfort to directly ask about adversity. Practitioner comfort peaked at 6 months with a decrease in comfort across most adversity types at 12 months which may reflect the challenge in overcoming practitioner fear.

Practitioner levels of comfort can be linked to their beliefs about their ability and their degree of fear or anxiety of directly asking about adversity. In numerous studies of practitioners screening for childhood adversities, practitioners report feeling uncomfortable to ask about adversities because of fear of a negative consequence including being judgemental or causing offense [19, 20, 46–49]. This is contrasted with families reporting that they are comfortable being asked about adversities [9, 10, 43, 50]. Fear of “opening pandora’s box” has been well described when practitioners ask about a range of adversities [48, 51, 52]. Sugg describes the “evils” released in opening Pandora’s box as the fear of offending, powerlessness, loss of control and the “tyranny of time” [51]. This is similar to the fear encountered in our study where practitioners were afraid of not being able to respond or of damaging the relationship with their families and thus limiting what they asked about. Another key practitioner fear is that they will not be able to “solve the problem”. In our study practitioner comfort improved with social connection and learning from one another. Social support enabled practitioners to become more comfortable to “hold” the problem rather than attempting to solve it. Improving practitioner comfort through decreasing their fear of a negative outcome is likely to improve behaviour change. In addition, future training and support needs to focus on supporting practitioners to manage multiple adversities as adversities cluster [7]. Practitioners prioritising which adversities to address in partnership with families may lead to increased agency and reduced fear of “opening Pandora’s box”.

Targeting psychological capability is critical in changing practice. Our study found a difference in practitioners who were choosing to change and those that did not. Practitioners who embraced reflective practice and were more mindful of seeking adversity were more likely to report an increase in directly asking about adversity. However, practitioners who saw themselves as experts and were less open to reflection reported that they did not see a need to change. Reflective practice is the process by which practitioners analyse a situation and assess what has been learnt from this experience and how this will change their actions in the future [45]. Reflection has been shown to make practitioners “more thoughtful” and to improve clinical practice [53]. Interestingly, reflection is not a behavioural determinant within the TDF although reflection is a considered an important determinant of behaviour [54]. Self-monitoring is a behavioural construct within the TDF ‘behavioural regulation’ and refers to the ability to monitor actions moment by moment and to examine learning and thinking processes [55]. Both reflection and self-monitoring have been found to be important skills for practitioners in having difficult clinical conversations but are usually poorly taught [55, 56]. Changing the emphasis of practitioner training from a focus on knowledge to an emphasis on developing reflective practice skills is likely to improve practitioner confidence and comfort to address childhood adversity.

We found the most important drivers of practice change were ‘social influence’ (e.g. social support and modelling), ‘knowledge’ (e.g. procedural knowledge), ‘belief about capabilities’ (e.g. self-confidence and perceived competence) and ‘behavioural regulation’ (e.g. making deliberate choices to change) and the main barriers were ‘environmental context and resources’ (e.g. time pressures and financial restraints) and ‘emotion’ (e.g. fear of negative outcome). This aligns with Mather et al. review of barriers and facilitators to behaviour change by primary care practitioners [57]. The most common domains identified across all studies were ‘environmental context and resources’, ‘knowledge’ and ‘social influences’ [57]. Mather et al. considered these the most important domains along with ‘skills’, ‘roles and responsibilities’ and ‘confidence in own ability’ [57]. This is supported by other reviews that have identified the most important domains for practice change being ‘environmental context and resources’, ‘social influence’, ‘knowledge’, ‘beliefs about ability’, and ‘beliefs about consequences’ [44, 58]. While there is a great deal of convergence of finding from all studies there are some notable exceptions. Interestingly while ‘emotion’ is frequently identified, authors were less likely to highlight this as an important domain however this was a key barrier to practice change in our study [57]. The importance of ‘social influence’ and ‘beliefs about capabilities’ across all studies emphasises the need to create opportunities for practitioner social support, coaching and building relationships. As Mathura found “relationships do matter, if you know somebody, and you trust them, it’s much more likely to do something that they are suggesting” [44]. 

### Strengths and limitations

This is the first study to use a theoretical framework to examine the determinants of practice change to understand the facilitators and barriers to practitioners identifying and responding to adversity. Additional strengths include a high response and retention rate and sampling practitioners from health and social care with a broad range of age and role experience.

This study has some limitations. Firstly, we had a small sample size of practitioners who were all from the same site which limits the generalisability of our outcomes. All the outcomes were by practitioner report. Practitioners have been shown to have a limited ability to do an accurate self-assessment thus the validity of their self-reported confidence and competence may be lower [59]. In addition, given the possibility of response bias where practitioners are more likely to report desirable behaviours, practitioner competence may be an overestimate. However, there is no gold standard measure of practitioner competence which necessitates the use of self-assessment in most health care settings [60]. Moreover, the quantitative data was collected with a specifically designed questionnaire so is not validated. Most practitioners were female, reflecting the social and health care workforce. It is not known if males may have responded differently.

Our focus was to improve practitioner identification and response to adversity, however, we found that practitioners were uncomfortable about directly asking about adversity. Having a focus on family resilience as well as adversity may have improved uptake by practitioners. Flanagan et al. instituted adversity screening paired with resilience screening and found that clinicians were more comfortable as it helped to “frame the adversity conversation” [10]. 

Finally, this project was part of an implementation of a health and social care hub which occurred just after the Covid-19 pandemic. While there were no state-wide lockdowns, there were periods of high infection rates which impacted on the health centre and the health practitioner’s workload and stress. The results from our study may be influenced by the impacts of the Covid-19 pandemic on practitioner’s wellbeing and feelings of burn out.

## Conclusions

We demonstrated that practitioners gained comfort to identify and confidence to respond to adversity through the implementation of multi-modal practice change processes delivered through an integrated Child and Family Hub. Changing practice takes more than just education and training. Purposeful provision of opportunities for social connection, building relationships and coaching to improve self-confidence and perceived competence were found to be important to realise practice change. Overcoming the “tyranny of time” which is in part, a function of the Australian healthcare fee-for-service funding model, would require a new approach to healthcare funding. If we are to improve outcomes for children, we need to better detect and respond to childhood adversity which will require a whole of practice change. The biggest danger is that we keep doing the same things and expect different outcomes.

### Electronic supplementary material

Below is the link to the electronic supplementary material.

Supplementary Material 1

## Data Availability

The datasets used and/or analysed during the current study are available from the corresponding author on reasonable request. The qualitative interview guide is found in supplementary files.

## Footnotes

1 Bulk billing refers to a payment option under the universal health insurance system in Australia (Medicare) where the cost of the consultation is billed to Medicare rather than to the patient.
