# Supplementary material for: Opening Pandora’s box - key facilitators of practice change in detecting and responding to childhood adversity - a practitioner perspective
Source: BMC Pediatr. 2024 Jul 18;24:461. doi: 10.1186/s12887-024-04918-5 (PMC11256471; doi:10.1186/s12887-024-04918-5)
Supplement: Supplementary file 1 — Supplementary Material 1 [file 12887_2024_4918_MOESM1_ESM.pdf]

Practitioner Interview Guide - 12-month Interview

Thank you for agreeing to take part in this interview. We want to understand your experiences working in the Child and Family Hub at Wyndham Vale/Marrickville.

I need to let you know that your participation in this research is entirely your choice. Also, it is entirely up to you whether you want to answer all of my questions. If at any stage you'd like to stop, take a break or don't want to answer any questions, just let me know. There are no right or wrong answers to questions – just your experiences and opinions, which are all valuable.

All the data that I collect from you will be treated in a confidential manner, so I'll be removing all information that identifies you from the transcript. You can ask for a copy of the transcript which will be sent to you.

Do you have any questions so far?

This interview will be recorded. You can ask to stop recording at any point during the interview. Are you happy to take part now? Are you happy for the interview to be recorded?

- start recording –

1. Can we begin with your role in the Child and Family Hub? Would you please share your experience of being in the Child and Family Hub over the past 12 months?

   Prompts:
   What have you enjoyed? What have you not enjoyed?
   What influenced your decision to take part in Child and Family Hub?

2. Can you tell me what you think the aims of the Child and Family Hub are?

The aim of the Child and Family Hub is to better identify and respond to childhood adversity. We aimed to do this through better integration of services and through training and support of practitioners.

3. What has been your experience of asking about adversity in families?

4. Has anything helped you identify adversity in families? (please explore what has helped? Any example/case?)

   Prompts:
   Thinking about the training, the lunchtime meetings, peer consultation/discussion, emails from Tony, the Hub handbook, being part of the bigger team, hearing from people who have experienced adversity – Did any of these things help you (please explore how and when: Any example/case?)

5. What has been your experience of responding to adversity in families? (please explore what kind of changes? Any example/case?)

6. Has anything helped you to respond to adversities in families?

Prompts:
Thinking about the training, the lunchtime meetings, peer consultation/discussion, emails from Tony, the Hub handbook, being part of the bigger team – did any of these things help you (please explore what kind of changes? Any example/case?)

7. What are the things than have stopped you making changes in identifying and responding to adversities in families?
   Prompts: These could be personal things like confidence or structural things such as funding (please explore, how and why)

8. Has being part of the Hub affected your enjoyment of your role in the at Wyndham Vale /Marrickville?
   a. If yes - can you say why?
   b. If no, can you say why not?

9. Has the Child and Family Hub approach changed how you engage with families around adversity?
   a) Yes - in what way (prompts - family partnership training impact, change in the language you use with families? Using more coaching?)
   b) No - can you say why not? (prompts - barriers to change e.g. confidence, skills, feel they are already engaging families well)

10. Let's talk about your experience of having lawyers / legal support in the Child and Family Hub.
    Prompts:
    What was good about having lawyers in the Child and Family Hub?
    What was the value of legal support to you as a practitioner or to your families? (examples)
    Did having legal support improve your confidence to identify legal issues or to link families into services. (Please explore why or why not?)
    Did having legal support cause any harms? (Please explore why or why not?)

11. Tell me about your experience of the well-being co-ordinator program (VIC) / care navigator (NSW) in the Child and Family Hub.
    Prompts:
    What was good about having a well-being co-ordinator (VIC) / care navigator (NSW) in the Child and Family Hub?
    What was the value of the well-being co-ordinator program (VIC) / care navigator (NSW) to you as a practitioner or to your families? (examples)
    Did having the well-being co-ordinator program cause any harms? (Please explore why or why not?)
    How do think this program could be improved?

12. Would you like to tell us about anything else required to strengthen the child and family hub approach to better address and respond to adversities for children and their families?

Thank you so much for talking with me today. I appreciate you taking the time to share your experiences with me.
